# Complete mitochondrial genome of *Plodia interpunctella* (Lepidoptera: Pyralidae)

**DOI:** 10.1080/23802359.2019.1710590

**Published:** 2020-01-14

**Authors:** Yu-Peng Wu, Tian-Juan Su, Bo He

**Affiliations:** a Taiyuan University of Science and Technology, Taiyuan, China;; b School of Life Sciences, Jinggangshan University, Jinggangshan, China;; c College of Life Sciences, Anhui Normal University, Wuhu, China

**Keywords:** *Plodia interpunctella*, complete mitogenome, Pyralidae

## Abstract

The *Plodia interpunctella* belongs to Pyralidae in Lepidoptera. The complete mitogenome of *P. interpunctella* was described in this study, which is typically circular duplex molecules and 15,403 bp in length, containing the standard metazoan set of 13 protein-coding genes, 22 transfer RNA genes, 2 ribosomal RNA genes, and an A + T-rich region. The gene order is the same as other lepidopterans. Except for *cox1* started with CGA, all other PCGs started with the standard ATN codons. Most of the PCGs terminated with the stop codon TAA, whereas *nad1* has the stop codon TAG, *nad4* has the incomplete stop codon T. The phylogenetic tree showed that *P. interpunctella* and *Corcyra cephalonica* are clustered into a clade Pyralidae.

*Plodia interpunctella* (Lepidoptera, Pyralidae) is a major pest of stored grain. If prevention and control are not timely, it will bring serious losses to agricultural production (Mbata and Osuji 1983). Since it is easy to get, like other insects, for example, *Drosophila melanogaster*, has been used as an experimental model insect. Many functional genes have been characterized (Zhu et al. 2000; Liu et al. 2015).

In this paper, the samples were collected by light trapping in Taiyuan city of China (37.833393, 112.666114) in July 2019, some of these specimens were immediately frozen at −80 °C on board for mitogenome analysis and others were preserved by spreading wings in the Herbarium of Institute of Plant Protection, Shanxi Academy of Agricultural Sciences, and their numbers is 20190605–20190610. Total genomic DNA was extracted from the tail tip using the Ezup pillar genomic DNA extraction kit (Sangon Biotech, Shanghai, China). The mitogenome was sequenced by Illumina Hiseq 4000. Gene annotation was performed and circularity was checked using the MITOS2 webserver (Bernt et al. 2013, http://mitos.bioinf.uni-leipzig.de/).

The mitochondrial genome of *P. interpunctella* has a total length of 15,403 bp (GenBank accession No. MN619781), consisting of 13 PCGs, 22 tRNA, 2 rRNA genes, and an A + T-rich region. As with other insect mitogenomes(Wu et al. 2016; Li et al. 2019), the major strand encodes a larger number of genes (9 PCGs and 14 tRNAs) than the minor strand (4 PCGs, 8 tRNAs, and 2 rRNA genes). Two rRNAs (16S rRNA and 12S rRNA) are located between tRNA-Leu(CUN) and tRNA-Val, and between tRNA-Val and the A + T-rich region, respectively. The 16S rRNA is 1332 bp in length and the 12S rRNA is 782 bp in length. The A + T-rich region is 320 bp long and located between 12S rRNA and tRNA-Met. The mitogenome contains 42.09% T, 38.19% A, 12.18% C, and 7.54% G, besides a high A + T content. All of the protein**-**coding genes have ATN as the start codon except for *cox1*, which starts with CGA. Eleven PCGs have the common stop codon TAA, *nad1* has the stop codon TAG, *nad4* has the incomplete stop codon T.

The phylogenetic position of *P. interpunctella* was inferred using sequences of the 13 PCGs of 21 species represented 20 families, besides a species *Anopheles gambiae* from Diptera (which was used as outgroup) (Figure 1). The sequences were aligned with MAFFT v7.2 software (Katoh and Standley 2013) and the evolutionary analyses were conducted with RAxML v8.2.10 (Stamatakis 2014) on the CIPRES Science Gateway (Miller et al. 2010). The GTRGAMMA model with ‘Let RAxML halt bootstrapping automatically’ was used. The phylogenetic tree was visualized using FigTree v1.4.4 (http://tree.bio.ed.ac.uk/software/figtree/). The result showed that *P. interpunctella* and *Corcyra cephalonica* are clustered into a clade Pyralidae.

**Figure 1.**
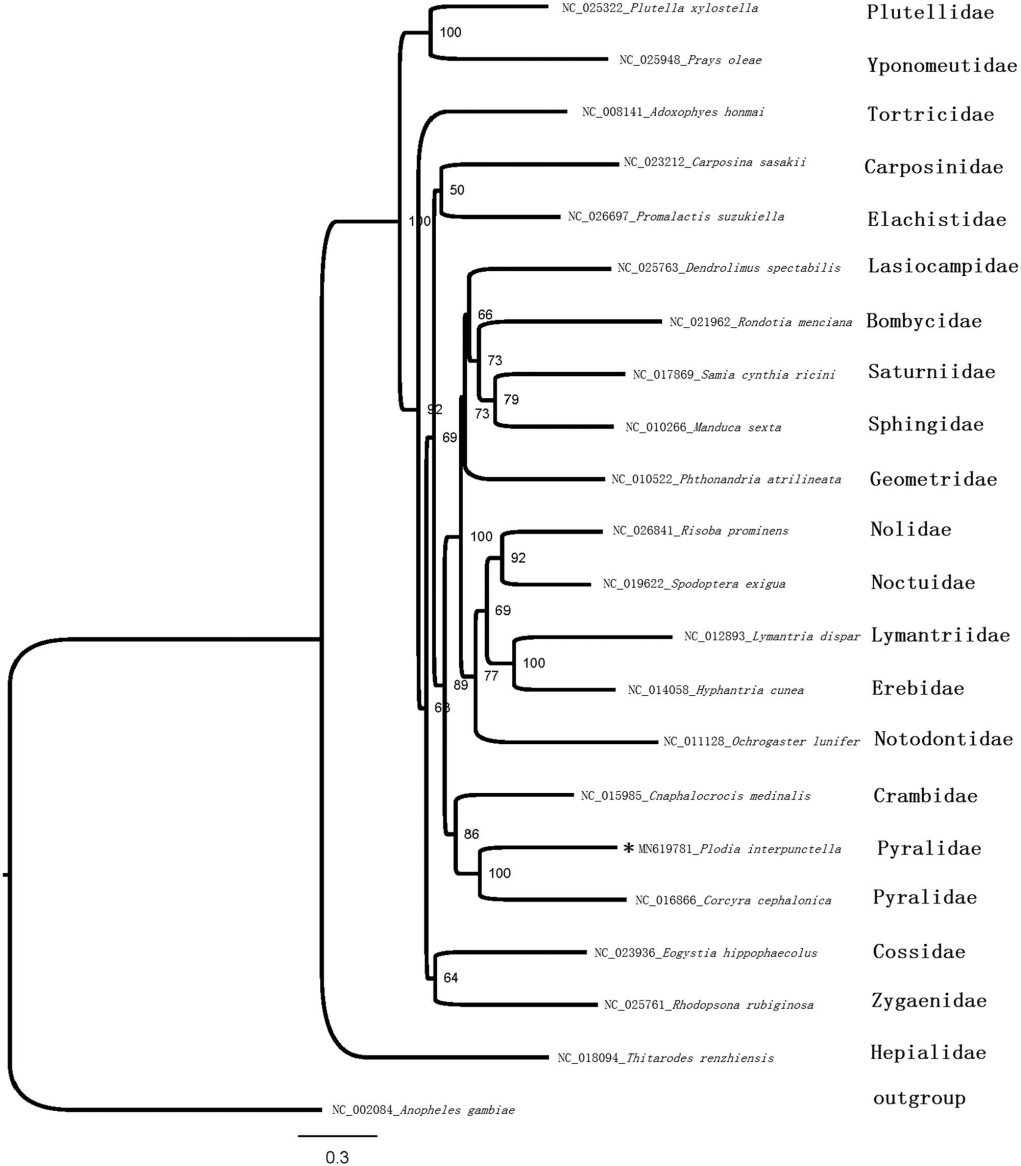
Maximum-likelihood tree of evolutionary relationships *P. interpunctella* based on the complete mitogenomes of 20 Lepidopteran moths.

## Nucleotide sequence accession number

The complete mitochondrial genome sequence of *P. interpunctella* was deposited in GenBank under the accession number MN619781.

## References

[CIT0001] Bernt M, Donath A, Juhling F, Externbrink F, Florentz C, Fritzsch G, Putz J, Middendorf M, Stadler PF. 2013. MITOS: improved de novo metazoan mitochondrial genome annotation. Mol Phylogenet Evol. 69(2):313–319. 22982435 10.1016/j.ympev.2012.08.023

[CIT0003] Katoh K, Standley DM. 2013. MAFFT multiple sequence alignment software version 7: improvements in performance and usability. Mol Biol Evol. 30(4):772–780. 23329690 10.1093/molbev/mst010 PMC3603318

[CIT0004] Li Y, Yang W, Zhuang J, Wei H, Liu X, Feng W, Yu H. 2019. Complete mitochondrial genome of *Phiaris dolosana* (lepidoptera: tortricidae). Mitochondr DNA Part B. 4(2):3198–3199. 10.1080/23802359.2019.1669083 PMC7707221 33365917

[CIT0005] Liu QN, Zhang HB, Jiang SH, Xuan FJ, Li CF, Zhang DZ, Zhou CL, Tang BP. 2015. The complete mitochondrial genome of *Eriocheir japonica sinensis* (Decapoda: Varunidae) and its phylogenetic analysis. Biochem Syst Ecol. 62:241–248.

[CIT0006] Mbata GN, Osuji F. 1983. Some aspects of the biology of *Plodia interpunctella* (Hübner) (Lepidoptera: Pyralidae), a pest of stored groundnuts in Nigeria. J Stored Prod Res. 19(3):141–151.

[CIT0007] Miller MA, Pfeiffer W, Schwartz T. 2010. Creating the CIPRES science gateway for inference of large phylogenetic trees. Proceedings of the Gateway Computing Environments Workshop (GCE); New Orleans, Louisiana: Institute of Electrical and Electronics Engineers (IEEE). p. 1–8.

[CIT0008] Stamatakis A. 2014. RAxML version 8: a tool for phylogenetic analysis and post-analysis of large phylogenies. Bioinformatics. 30(9):1312–1313. 24451623 10.1093/bioinformatics/btu033 PMC3998144

[CIT0009] Wu YP, Zhao JL, Su TJ, Luo AR, Zhu CD. 2016. The complete mitochondrial genome of *Choristoneura longicellana* (Lepidoptera: Tortricidae) and phylogenetic analysis of Lepidoptera. Gene. 591(1):161–176. 27390085 10.1016/j.gene.2016.07.003

[CIT0010] Zhu YC, Kramer KJ, Oppert B, Dowdy AK. 2000. cDNAs of aminopeptidase-like protein genes from *Plodia interpunctella* strains with different susceptibilities to *Bacillus thuringiensis* toxins. Insect Biochem Mol Boil. 30(3):215–224. 10.1016/s0965-1748(99)00118-6 10732989

